# Effects of Exercise on Neural Changes in Inhibitory Control: An ALE Meta-Analysis of fMRI Studies

**DOI:** 10.3389/fnhum.2022.891095

**Published:** 2022-06-24

**Authors:** Jinlong Wu, Wen Xiao, Joanne Yip, Li Peng, Kangyong Zheng, Obed Takyi Bentil, Zhanbing Ren

**Affiliations:** ^1^School of Physical Education, Shenzhen University, Shenzhen, China; ^2^Institute of Textiles and Clothing, The Hong Kong Polytechnic University, Hong Kong, China; ^3^College of Physical Education, Southwest University, Chongqing, China; ^4^Department of Sport Rehabilitation, Shanghai University of Sport, Shanghai, China; ^5^Civil and Environmental Engineering Department, The Hong Kong Polytechnic University, Kowloon, Hong Kong SAR, China

**Keywords:** exercise, inhibitory control, meta-analysis, activation likelihood estimation, fMRI

## Abstract

It is widely known that exercise improves inhibitory control; however, the mechanisms behind the cognitive improvement remain unclear. This study analyzes the extant literature on the neuronal effects of exercise on inhibitory control functions. We searched four online databases (Pubmed, Scopus, PsycINFO, and Web of Science) for relevant peer-reviewed studies to identify eligible studies published before September 1, 2021. Among the 4,090 candidate studies identified, 14 meet the inclusion criteria, and the results of 397 participants in these 14 studies are subsequently analyzed. We quantify the neural effects on the entire brain by using GingerALE software and identify 10 clusters of exercise-induced neuronal with either increases/decreases in the superior temporal gyrus (BA 22), precuneus (BA 7), superior frontal gyrus (BA 10), cuneus (BA 19), precuneus (BA 19), caudate, posterior cingulate (BA 19), middle temporal gyrus (B 37), parahippocampal gyrus (BA 30), precentral gyrus (BA 6). Meta-analytic coactivation map (MACM) showed that multiple functional networks overlap with brain regions with activation likelihood estimation (ALE) results. We propose the effect of exercise on neural activity is related to inhibitory control in the extended frontoparietal, default mode network (DMN), visual network, and other pathways. These results provide preliminary evidence of the neural effects of exercise on inhibitory control.

## Introduction

Inhibitory control or inhibition is defined as suppressing prepotent responses to goal-irrelevant stimuli and contributes to anticipation, planning, and goal setting. Inhibitory control is one of the three core executive functions of the brain (Aron, 2007; Liang et al., 2021b). People who suffer from impaired inhibitory control have a lower quality of life and develop health problems and diseases which applies to healthy and sick people (Liang et al., 2021a). Meanwhile, impaired inhibitory control could be a hallmark feature of several neuropsychological DSM-5 disorders including attention-deficit/hyperactivity disorder (ADHD; Crosbie et al., 2008; Bari and Robbins, 2013), bipolar disorder (Hidiroglu et al., 2015), schizophrenia (Enticott et al., 2008; Hughes et al., 2012), and substance use disorders (Liao et al., 2014; Lee et al., 2015). Although the disorders listed here are primarily characterized by difficulties in controlling behavior (Brady et al., 2011; Sofuoglu et al., 2013; Baumeister et al., 2018), they are also found in normal cognitive aging (Coxon et al., 2012; Smittenaar et al., 2015). Thus, to understand the risk of developing these disorders and contribute to current prevention and treatment measures, an analysis of the neural underpinnings of inhibitory control is essential and timely.

Physical activity has garnered significant attention as a potentially effective method for elevating cognitive function and improving brain health throughout life (Loprinzi et al., 2013; Ji et al., 2021). Meta-analyses of healthy participants (Li et al., 2020; Amatriain-Fernandez et al., 2021; Chen et al., 2021), autism spectrum disorder (ASD; Liang et al., 2021b), mild cognitive impairment (MCI; Biazus-Sehn et al., 2020) as well as ADHD patients (Liang et al., 2021a) have shown that exercise has a positive effect on inhibitory control in individuals. Neuroimaging studies have also demonstrated the inhibitory control mechanisms of the brain by exploring the relationship between exercise and brain region activation during specific tasks (e.g., flanker, go/no-go, and Stroop tasks). Previous studies reported that the “cognitive control network” actively coordinates multiple brain regions, such as the frontal cortex (including the anterior cingulate cortex), parietal cortex, motor regions, and cerebellum (Niendam et al., 2012; Akatsuka et al., 2015; Chu et al., 2015). The effects of exercise on functional changes in inhibitory control are not apparent. Indeed, some studies report an increase in the activation of the prefrontal and parietal lobes following exercise intervention (Mehren et al., 2019c), whereas others reported less activation in the frontal and temporal lobes during similar inhibitory-based tasks (Krafft et al., 2014; Hsu et al., 2018).

The last few years have seen the introduction of several tools for performing a meta-analysis of data obtained from brain imaging research, allowing for the quantitative integration of findings from different studies. Activation likelihood estimation (ALE) is a relatively way to estimate the probability that at least one activation focus from a set of experiments lies at the location of a specific voxel, using Gaussian assumptions of spatial uncertainty (Turkeltaub et al., 2002) This study, using the ALE method, aimed to explore the overall neural changes in inhibitory control associated with exercise. We hypothesized the critical regions of exercise-induced inhibitory control are related to several frontal and parietal cortex brain areas. To validate ALE results whether overlap the brain networks to related frontoparietal or other brain networks, we applied a meta-analytic co-activation model (MACM) approach using activation clusters as regions of interest (ROIs) from our ALE results.

## Method

The meta-analysis in this study is completed and reported by the Preferred Reporting Items for Systematic Reviews and Meta-Analyses Statement (PRISMA). The protocol was registered as trial registration number CRD42021285736 under the International Prospective Register of Systematic Reviews (PROSPERO).

### Search Strategy

Four electronic databases (Pubmed, Scopus, PsycINFO, and Web of Science) were searched from their inception to September 1, 2021, to identify all published studies on functional magnetic resonance imaging (fMRI) that investigate the impact of exercise on the activation of different brain areas during inhibitory control tasks. The initial search used three key terms: physical activity or exercise, inhibitory control, and fMRI. We also hand-searched recent systematic reviews and meta-analyses to identify potential studies (Liang et al., 2021a; Yu et al., 2021). The search was limited to English-language results and human subjects. The reference lists of included studies were manually reviewed for relevant articles that were captured through the database searches. The detailed keyword search strategy is presented in the Appendix Table.

### Study Selection

The screening for relevant studies was conducted in accordance with the PICOS (participants, intervention, comparisons, outcomes, and study design) principles. The participants included individuals of all ages and pathologies. The studies must have investigated the effect of exercise on inhibitory control and examined pre/post-intervention with at least one group assigned to physical activity/exercise intervention. During fMRI scanning, studies must have assessed brain activation patterns *via* completed inhibitory control tasks (e.g., go/no-go, stroop, flanker, or Simon tasks). For inclusion, retrievable data in standard Talairach or Montreal Neurologic Institute (MNI) space was also required. Finally, the chosen studies included randomized controlled trials (RCTs) and non-randomized controlled trial studies (NRCTs) published in peer-reviewed journals.

Two reviewers independently conducted the multi-step search process based on these selection criteria and screened the studies based on their title and abstract. Full-length texts were eventually used to identify eligible articles. If consensus could not be reached, a third reviewer made the final decision after discussion with the two reviewers.

### Data Extraction

A standardized data extraction form was developed to extract relevant data from each study, including the bibliographic details (author and year), participant characteristics (sample size, sex, and age range), intervention components (intervention design and duration), fMRI task, software used, and active results/foci.

### Quality Assessment

The Physiotherapy Evidence Database (PEDro) scale, a reliable and valid instrument for assessing the methodological quality of studies that focus on the effects of physical activity on cognitive functions, was used to determine the methodological quality (Sherrington et al., 2000). This scale includes 11 rating criteria including eligibility, randomization, allocation, blinding (subjects and experimenter), intention-to-treat, between-group comparison, and point measures. The methodological criteria were scored as Yes (one point), No (zero points) or Do not know (zero points). The PEDro score of each selected study served as an indicator of the methodological quality (<4 = poor; 4–5 = fair; 6–8 = good; and 9–10 = excellent).

The fMRI quality was determined from a set of guidelines for the standardized reporting of the fMRI studies and used to assess the fMRI study design/reporting quality and quality of the fMRI data of each included study (Poldrack et al., 2008). The fMRI form has 8-item rating criteria for eligibility, the experimental design, handedness and gender of participants, explanation for rejected data, details of the imaging parameters, software analysis method and package used, motion correction method during pre-processing, multiple comparison correction, and detailed description of the first and second level analyses.

### Data Analysis

The ALE is a reliable quantitative method for coordinate-based meta-analyses to identify brain activation during cognitive functions after exercise (Laird et al., 2005). In this study, GingerALE v2.3.6 (http://www.brainmap.org) software is used to analyze the data. A statistical threshold of uncorrected *p* < 0.001 and a minimum cluster size of 100 mm^3^ were used (Meng et al., 2020). The ALE maps were imported into Mango Version 4.1 (http://ric.uthscsa.edu/mango/mango.html) software and overlaid on an anatomical template in MNI space for visualization. The effect of exercise on behavioral performance is not examined due to missing data and changes in an inhibitory control task that resulted in the effect magnitude being inestimable.

Additionally, to obtain the MACM based on the ALE results, we followed the procedure proposed by Robinson et al. (2010) as implemented in NeuroSynth (http://neurosynth.org/; Yarkoni et al., 2011). The activation clusters as seed ROIs were separately entered into NeuroSynth to evaluate the MACM. In brief, the software searches among more than 11,000 fMRI studies (totaling 413,429 activations in the MNI152 coordinate space) those reporting activation in a spherical seed (6 mm) around the searched coordinates. The identified co-activations are then pooled together to form the MACM output that is corrected for multiple comparisons (*p* < 0.01 false discovery rate; FDR as provided in NeuroSynth). Namely, a z-score is assigned to each voxel, representing the strength of the association between a given voxel and the seed coordinates.

## Results

### Study Selection

**Figure 1:**
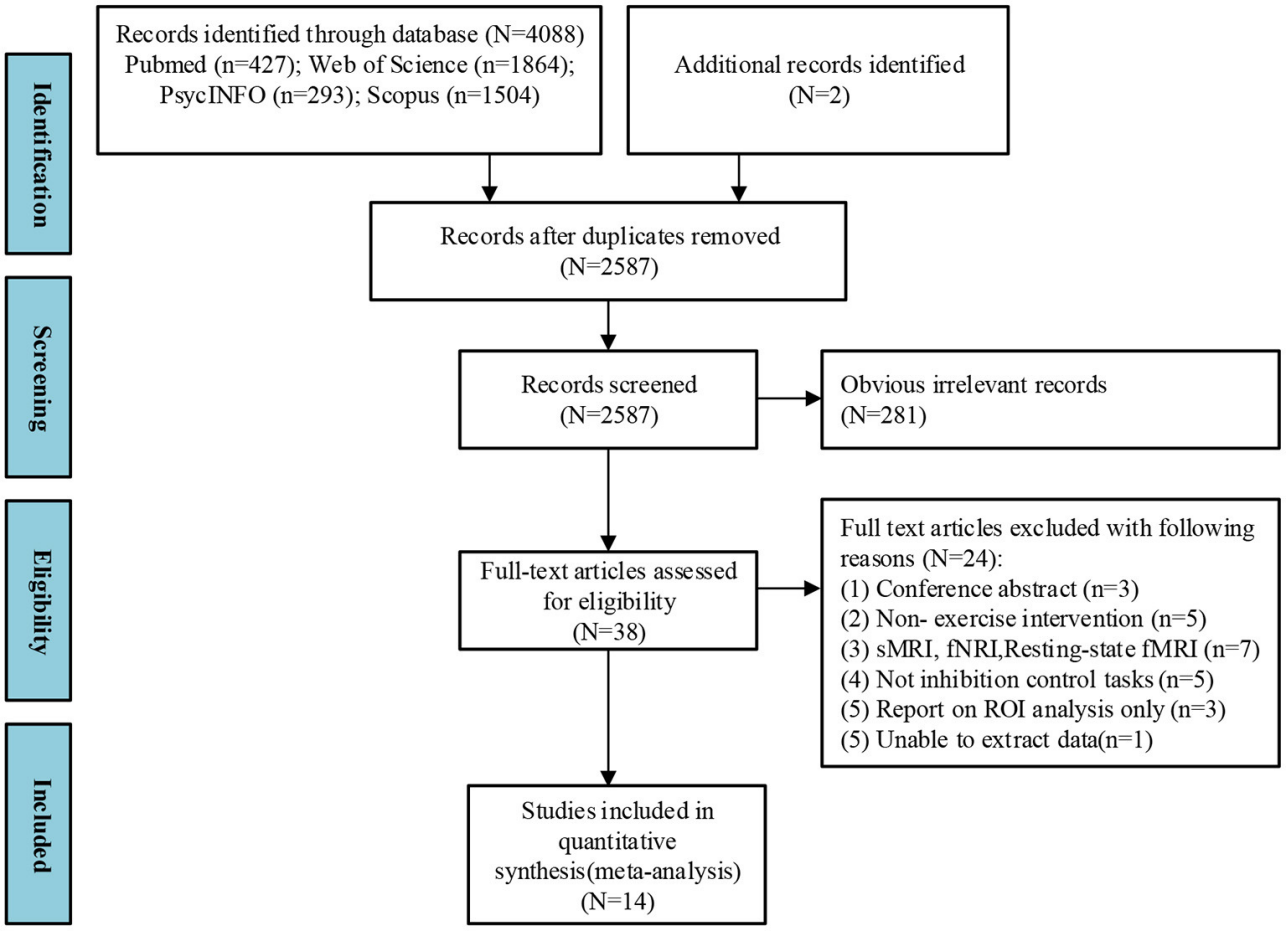
PRISMA flow diagram of selected studies. Multi-step search process shows the number of studies screened and those that meet the inclusion criteria.

Four thousand and eighty-eight studies were identified from the four databases, and two studies were identified from other systemic reviews. Included studies were chosen after thoroughly screening the titles, abstracts, and full text. Fourteen studies were identified for the meta-analysis (Figure 1).

### Study Characteristics

Table 1 lists the characteristics of the 14 studies that involved 397 participants. Six studies (Mehren et al., 2019a,b,c; Won et al., 2019; Cui et al., 2020; Meng et al., 2020) utilized acute exercise paradigms and eight studies (Colcombe et al., 2004; Liu-Ambrose et al., 2012; Krafft et al., 2014; Sachs et al., 2017; Hsu et al., 2018; Martinsen et al., 2018; Pensel et al., 2018; Wu et al., 2018) utilized chronic exercise routines. Three neurocognitive tasks (including flanker, stroop, and go/no-go tasks) were used to assess inhibitory control in participants across the studies.

**Table 1:** Descriptive characteristics of included studies.

**References**	**Design**	**Participants**	**Sample male/ female**	**Age (years old)**	**Intervention for exercise group**	**Duration**	**fMRI task**	**Software**	**Active Results/Foci**
Colcombe et al. (2004)	RCT	Older adults	M: 11 F: 18	65.6 ± 5.66	Walking	40–45 min/session * 3 days/week * 6 months	Flanker task	SPM99	Pre>Post/ 0 Post>Pre/ 3
Liu-Ambrose et al. (2012)	RCT	Senior women	F: 52	Rt1:69.7 ± 2.8 Re2:68.9 ± 3.2 Bat: 69.3 ± 3.0	Resistance exercise	Rt1:1 days/week *52 months Re2:2 days/week *52 months Bat: 2 days/week *52 months	Eriksen flanker task	FEAT and FSL	Pre>Post/ 0 Post>Pre/ 12
Krafft et al. (2014)	RCT	Overweight children	M: 7 F: 17	9.7 ± 0.8	Tag and jump rope	405 min/session * 7 days/week * 8 months	Flanker task	AFNI	Pre>Post/ 6 Post>Pre/ 4
Metcalfe et al. (2016)	nRCT	Adolescents with bipolar disorder	M: 13 F: 17	16.8 ± 1.4	Recumbent bicycle-ergometer	27 min	Go-no-go task	FSL	Pre>Post/ 5 Post>Pre/ 3
Sachs et al. (2017)	nRCT	Children	M: 5 F: 8	8.85	Soccer and swimming	60 min/session * 2 or 3 days/week * 5 years	Color-word stoop task	FSL	Pre>Post/ 1 Post>Pre/ 0
Martinsen et al. (2018)	nRCT	Fibromyalgia	F: 19	49.6	Resistance exercise	60 min/session * 2 days/week * 15 weeks	Color-word stoop task	SPM8	Pre>Post/ 0 Post>Pre/ 3
Hsu et al. (2018)	RCT	SIVCI	M: 4 F: 6	M:71.1 ± 8.8 F: 73.5 ± 7.9	Walking	60 min/session * 3 days/week * 6 months	Flanker task	FSL	Pre>Post/ 16 Post>Pre/ 0
Pensel et al. (2018)	nRCT	Order adults	M:23	M:49.00 ± 5.32	Running	60 min/session * 3 days/week * 6 months	Flanker task	SPM8	Pre>Post/ 0 Post>Pre/ 22
Wu et al. (2018)	RCT	Order adults	16	64.9 ± 2.8	Taichi	60 min/session * 3 days/week * 12 weeks	Stoop task	SPM12	Pre>Post/ 0 Post>Pre/ 5
Mehren et al. (2019a)	nRCT	Adults	MI:M:16 F:16 HI:M:15 F:16	MPA:29.3 ± 8.5 MVPA:28.6 ± 7.7	Cycling and Hiit	30 min	Go-no-go task	SPM12	Pre>Post/ 0 Post>Pre /5
Mehren et al. (2019c)	nRCT	Adult patients with ADHD	M: 20 F: 3	31.4 ± 9.6	Cycling	30 min	Go-no-go task	SPM12	Pre>Post/ 0 Post>Pre/ 3
Mehren et al. (2019b)	nRCT	Adult patients with ADHD	M: 16 F: 4	29.9 ± 9.5	Cycling	30 min	Flanker task	SPM12	Pre>Post/ 0 Post>Pre/ 5
Won et al. (2019)	nRCT	Older adults	M: 8 F: 24	66.2 ± 7.3	Cycling	30 min	Eriksen flanker task	AFNI	Pre>Post/ 2 Post>Pre/ 9
Cui et al. (2020)	nRCT	Female college students	F: 43	HF:20.32 ± 0.75 LH:20.35 ± 0.61	Cycling	30 min	Stoop task	SPM23	Pre>Post/ 0 Post>Pre/ 10

*RCT, randomized control trial; nRCT, non-randomized control trial; SIVCI, subcortical ischemic vascular cognitive impairment; ADHD, attention deficit hyperactivity disorder; BAT, twice-weekly balance and tone training; RT1, once-weekly resistance training; RT2, twice-weekly resistance training; HT, high-fit group; LF, low-fit group; MI, moderate intensity; HI, high intensity; HITT, high-intensity interval training*.

### Quality Assessment

Table 2 shows the methodological quality of the studies using the PEDro scale. The total scores range from 6 to 9 (*M* = 6.71). Notably, none of the studies reported blinded data due to the difficulties of using blinded subjects, therapists, and assessors in an exercise intervention. The studies that failed to obtain points in other criteria due to their study design include lack of eligibility (*n* = 1), random allocation (*n* = 9), and concealed allocation (*n* = 10).

**Table 2:** Methodological quality assessment of included studies.

**References**	**Eligibility criteria**	**Random allocation**	**Concealed allocation**	**Similar at baseline**	**Subject blinded**	**Therapist blinded**	**Assessor blinded**	**Dropout**	**Intention-to-treat analysis**	**Between-group comparison**	**Points measures**	**Total score**	**Overall study quality**
Colcombe et al. (2004)	0	0	0	1	0	0	0	1	1	1	1	5	Good
Liu-Ambrose et al. (2012)	1	1	1	1	0	0	1	1	1	1	1	9	Excellent
Krafft et al. (2014)	1	1	1	1	0	0	0	1	1	1	1	8	Good
Metcalfe et al. (2016)	1	0	0	1	0	0	0	1	1	1	1	6	Good
Sachs et al. (2017)	1	0	0	1	0	0	0	1	1	1	1	6	Good
Martinsen et al. (2018)	1	0	0	1	0	0	0	1	1	1	1	6	Good
Hsu et al. (2018)	1	1	1	1	0	0	0	1	1	1	1	8	Good
Pensel et al. (2018)	1	0	0	1	0	0	0	1	1	1	1	6	Good
Wu et al. (2018)	1	1	0	1	0	0	1	1	1	1	1	8	Good
Mehren et al. (2019a)	1	0	0	1	0	0	0	1	1	1	1	6	Good
Mehren et al. (2019c)	1	0	0	1	0	0	0	1	1	1	1	6	Good
Mehren et al. (2019b)	1	0	0	1	0	0	0	1	1	1	1	6	Good
Won et al. (2019)	1	0	0	1	0	0	0	1	1	1	1	6	Good
Cui et al. (2020)	1	1	1	1	0	0	0	1	1	1	1	8	Good

*Yes = 1; No or Do not know = 0*.

Table 3 shows the fMRI quality assessment for all of the studies from a set of guidelines for the standardized reporting of fMRI studies. Other criteria were lacking in at least one reporting guideline, particularly in the scan rejection mentioned (*n* = 13), scan rejection reason (*n* = 14), the method for motion correction (*n* = 7), volume acquired per session (*n* = 14), and clear descriptions of the first (*n* = 8) and second level contrasts (*n* = 7).

**Table 3:** fMRI quality assessment of included studies.

**References**	**fMRI design**	**Sample handedness reported**	**Sample gender reported**	**Scan rejection mentioned**	**Scan rejection reason**	**Volume acquired per session**	**Software package specified**	**Method for motion correction described**	**Method for multiple comparison correction described**	**Type of correction applied**	**First level contrasts described**	**Second level contrasts described**
Colcombe et al. (2004)	1	0	0	0	0	0	0	0	0	Voxel wise	0	1
Liu-Ambrose et al. (2012)	1	1	1	0	0	0	1	1	0	Cluster	1	1
Krafft et al. (2014)	1	1	1	1	0	0	1	0	1	Cluster	0	0
Metcalfe et al. (2016)	1	0	1	0	0	0	1	1	0	Unclear	1	0
Sachs et al. (2017)	1	1	1	0	0	0	1	1	0	Cluster	0	0
Martinsen et al. (2018)	1	0	1	0	0	0	1	0	1	Unclear	1	1
Hsu et al. (2018)	1	0	1	0	0	0	1	1	0	Cluster	0	1
Pensel et al. (2018)	1	1	1	0	0	0	1	0	1	Voxel wise	1	1
Wu et al. (2018)	1	1	1	0	0	0	1	1	1	Voxel wise	1	1
Mehren et al. (2019a)	1	1	1	0	0	0	1	0	1	Voxel wise	0	0
Mehren et al. (2019c)	1	1	0	0	0	0	1	0	1	Voxel wise	0	0
Mehren et al. (2019b)	1	1	1	0	0	0	1	0	1	Voxel wise	0	0
Won et al. (2019)	1	1	1	0	0	0	1	1	0	Voxel wise	0	0
Cui et al. (2020)	1	1	1	0	0	0	1	1	1	Voxel wise	1	1

*Yes = 1; No or do not know = 0*.

### The Overall Analysis of Activity Results

**Figure 2:**
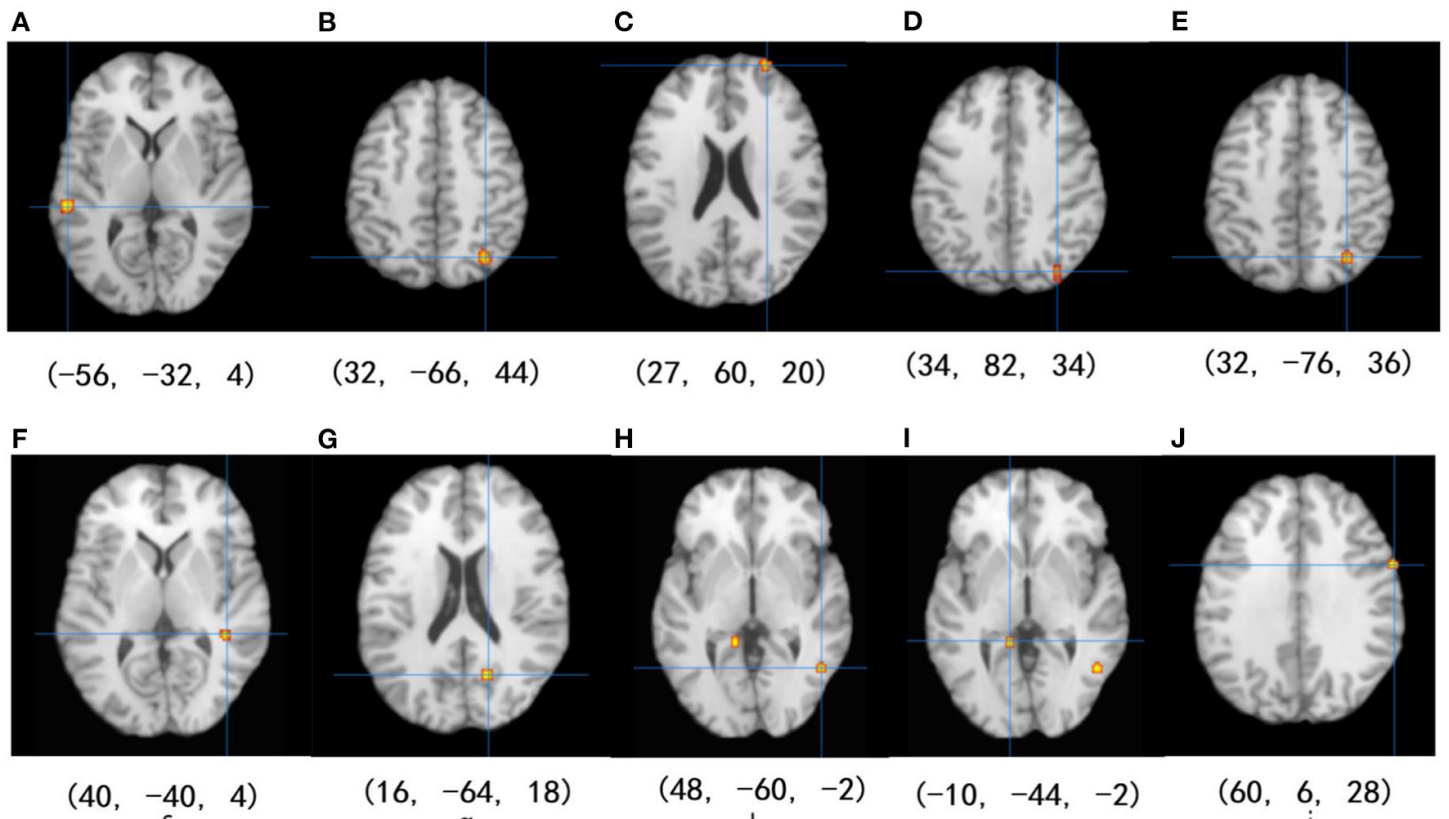
Results of the ALE meta-analysis show increased and reduced activation for inhibitory control in the EGs compared with CGs. Uncorrected *p* < 0.001 and cluster size >100 mm^3^ (from **A–D**: activation is increased, and from **E–J**: activation is decreased).

Among the 14 studies that assessed brain activation during inhibitory control tasks after exercise, the exercise groups (EGs) show increased brain activation compared to the control groups (CGs) in six clusters: (1) superior temporal gyrus (BA 22), (2) precuneus (BA 7 and BA 19), (3) superior frontal gyrus (BA 10), and (4) cuneus (BA 19). Five regions showed reduced brain activation: (1) caudate gray matter, (2) posterior cingulate cortex (BA 31), (3) middle temporal gyrus (BA 37), (4) parahippocampal gyrus, and (5) precentral gyrus (BA 6), as shown in Table 4 and Figure 2.

**Table 4:** ALE clusters derived from inhibitory control task in overall analysis.

**Cluster**	**Region**	**Brodmann area**	**x**	**y**	**z**	**ALE extrema**	***p***
**Activation increases**							
1	L Superior temporal gyrus	BA 22	−56	−32	4	0.015	<0.001
2	R Precuneus	BA 7	32	−66	44	0.012	<0.001
3	R Superior frontal gyrus	BA 10	27	60	20	0.012	<0.001
4	R Cuneus	BA 19	34	82	34	0.010	<0.001
5	R Precuneus	BA 19	32	−76	36	0.009	<0.001
**Activation decreases**							
1	R Caudate	–	40	−40	4	0.009	<0.001
2	R Posterior cingulate	B31	16	−64	18	0.009	<0.001
3	R Middle temporal gyrus	BA 37	48	−60	−2	0.008	<0.001
4	L Parahippocampal gyrus	BA 30	−10	−44	−2	0.008	<0.001
5	R Precentral gyrus	BA 6	60	6	28	0.008	<0.001

*R, right; L, left*.

### Coactivation Maps

Coactivation maps were plotted after the MACM analysis, as shown the Table 5. To understand the correspondence between the obtained coactivation maps and the brain functional connectivity networks, we compared it with the cortical parcellation atlas built by Yeo et al. (2011) on 1,000 healthy young subjects. We found that multiple functional networks, including the frontoparietal network, visual network, default mode network, and attention network, overlap with brain regions with ALE results, although this is a subjective judgment.

**Table 5:** The results of co-activation maps.

**Cluster**	**x**	**y**	**z**		**L**	**R**
L Superior temporal gyrus	−56	−32	4	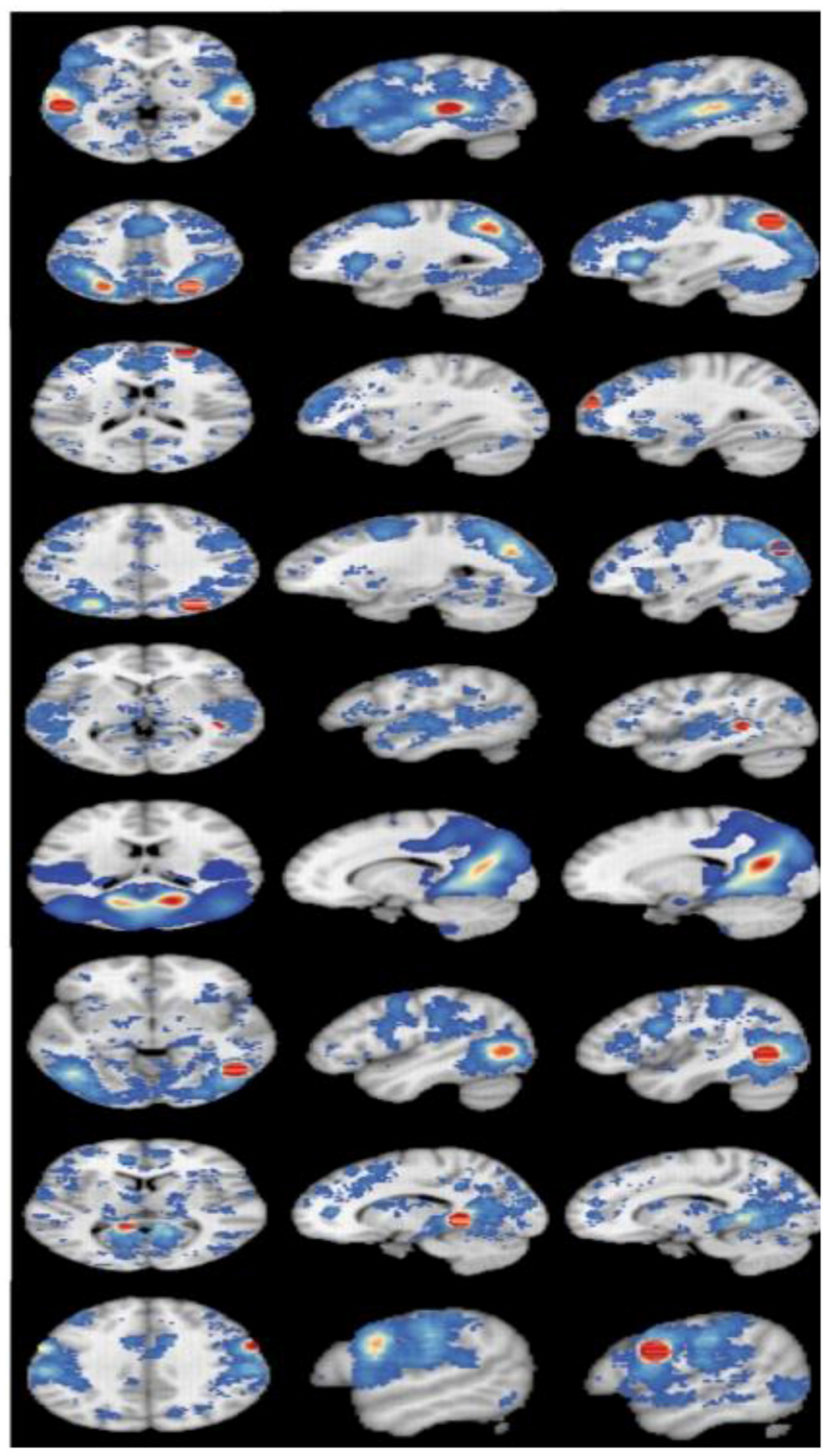		
R Precuneus	32	−66	44			
R Superior frontal gyrus	27	60	20			
R Precuneus	32	−76	36			
R Caudate	40	−40	4			
R Posterior cingulate	16	−64	18			
R Middle temporal gyrus	48	−60	−2			
L Parahippocampal gyrus	−10	−44	−2			
R Precentral gyrus	60	6	28			

*R, right; L, left*.

## Discussion

The primary aim of the ALE meta-analysis in this study is to offer the first quantitative summary of the effect of exercise on increased and decreased neural activity of different brain areas during inhibitory control tasks. We identified five clusters of increased neural activity [left superior temporal gyrus, right precuneus (BA 7 and BA 19), right superior frontal gyrus, and right cuneus] and five clusters of diminished neural activity (left parahippocampal, right posterior cingulate, right middle temporal and right precentral gyrus, and right caudate) following exercise. MACM analysis suggested that inhibitory control involved multiple functional networks. These findings may help identify the underlying mechanisms of increased inhibitory control that are exercise-induced.

Previous work found that the neurophysiological mechanism that controls movement is located in the prefrontal cortex (Niendam et al., 2012; Ardila et al., 2018). Our finding shows that there are changes in brain activity in the superior frontal and precentral gyrus of the frontal lobe. It should be noted that both of these regions are responsible for motor control (Niendam et al., 2012; Ardila et al., 2018).

The superior frontal gyrus is also part of the prefrontal cortex, which is responsible for cognitive control functions. This is closely connected with high-level cognitive functions such as interference inhibition, conflict solving, and selective attention (Cabeza and Nyberg, 2000; Baym et al., 2008). The precentral gyrus is part of the supplementary motor area (SMA), which is essential for cognitive control as found in previous studies of concern, especially when motor movements need to be inhibited (Nachev et al., 2008; Aron, 2010).

Our findings showed one change in brain activity in the parietal lobe is the precuneus. The precuneus region is an essential area for attention selection and response to conflict (Indovina and Macaluso, 2004). Previous research found that it plays a pivotal role in the prefrontal-parietal circuit when performing inhibitory tasks (Garavan et al., 2002; Mehren et al., 2019c). Thus, this study concludes that exercise is closely related to connectivity in the frontoparietal network. However, more studies are needed to verify this conclusion.

Besides, the finding showed that three regions' activity changed, including the superior temporal gyrus, middle temporal gyrus, and caudate in the temporal lobe during inhibitory control tasks. It is worthy to note that the superior temporal sulcus typically provides the amygdala with visual information that contributes to identifying the affective or motivational significance of visually perceived objects (Arzimanoglou et al., 2005). An aerobic intervention experiment by Hsu et al. (2018) showed that aerobic intervention alters the activation of the superior temporal gyrus in patients with cognitive impairment and pointed out that there is an apparent correlation between the activation of the superior temporal gyrus and the reaction speed in the go/no-go task. Liu-Ambrose et al. found that after 52 elderly subjects received resistance training twice a week for 12 months, the pathway extending from the left anterior middle temporal gyrus and the left anterior insula to the lateral orbitofrontal cortex was activated during the flanker task (Liu-Ambrose et al., 2012).

It has been proven that the caudate is one of the primary input nuclei receiving inputs from the prefrontal cortex and transferring information to the basal ganglia (Kunishio and Haber, 1994; Haber et al., 2000; Nakahara et al., 2002). The dorsolateral prefrontal cortex-caudate circuit was also shown to be involved in proactive inhibition *via* the indirect pathway (Jahfari et al., 2011). We speculate that exercise effectively activates these pathways in response to inhibitory tasks. However, additional experiments are required for verification.

Finally, we found posterior cingulate gyrus and parahippocampal gyrus have activation changes, and these two regions both belong to the limbic structures. The posterior cingulate gyrus is close to the limbic system and belongs to the cortical region functioning as a gateway between the limbic system and the midbrain and diencephalon (Li et al., 2017). Since the posterior cingulate gyrus and precuneus are identified here, we hypothesize that exhibited hyper-connectivity (i.e., stronger positive connectivity) is shown in the default mode network (DMN) regions during inhibitory control tasks which is consistent with findings in previous studies (Yu et al., 2021). Furthermore, the degree of coupling between the DMN and the frontal-parietal network positively correlates with overall cognitive function. Therefore, the DMN, which is the main component for the stop signal in inhibitory tasks is particularly significant for the impact of physical exercise on inhibitory control ability.

The parahippocampal gyrus is an active region in the limbic system, and exercise intervention appears to strengthen their neuronal excitability, increase the volume of white matter/gray matter, and positively change the production of brain-derived neural factors (Li et al., 2017; Loprinzi, 2017; Muller et al., 2017; Ji et al., 2021). Previous research has validated the effect of exercise on the plasticity of the parahippocampal gyrus, which plays a crucial role in maintaining memory function and aids in spatial information processing and object recognition (Brown and Aggleton, 2001; Raslau et al., 2015). We guess that the parahippocampal gyrus of spatial information processing and object recognition functions are also likely the underlying reason(s) that exercise improves cognitive control function.

There are some limitations to this study. First, only 14 studies are included, which is a smaller sample size. As a result, a subgroup analysis of the exercise intensity and type of exercise is challenging. Second, this study contains different inhibitory control tasks; although the flanker, go-no-go, and stroop tasks are all standard tasks for measuring inhibitory control, the components are slightly different.

## Conclusions

Neural mechanisms increase exercise-induced inhibitory control by changing the activation of single and multiple brain regions. We guess that critical areas mediate inhibitory control and are associated with the frontoparietal, visual network, DMN, and other pathways. However, due to the lenient threshold used in the ALE analysis, further research with more rigorous methods is required to link exercise to neuroplasticity changes with respect to inhibitory control.

## Data Availability Statement

The raw data supporting the conclusions of this article will be made available by the authors, without undue reservation.

## Author Contributions

JW and ZR were responsible for the conceptualization, investigation, and hypothesis of the research. JW and WX conducted systematic search, data extraction, quality assessment, and data analyses. JY, KZ, LP, OT, and ZR reviewed and edited the initial draft and revisions. All authors contributed to the article and approved the submitted version.

## Funding

The article was supported by Research Foundation for Young Teacher of Shenzhen University [grant number QNJS0274]; High-level Scientific Research Foundation for the Introduction of Talent of Shenzhen University [grant number RC00228]; and Natural Science Featured Innovation Projects in Ordinary Universities in Guangdong Province [grant number 2021KTSCX297].

## Conflict of Interest

The authors declare that the research was conducted in the absence of any commercial or financial relationships that could be construed as a potential conflict of interest.

## Publisher's Note

All claims expressed in this article are solely those of the authors and do not necessarily represent those of their affiliated organizations, or those of the publisher, the editors and the reviewers. Any product that may be evaluated in this article, or claim that may be made by its manufacturer, is not guaranteed or endorsed by the publisher.

## References

[B1] Akatsuka K. Yamashiro K. Nakazawa S. Mitsuzono R. Maruyama A. (2015). Acute aerobic exercise influences the inhibitory process in the go/no-go task in humans. Neurosci. Lett. 600, 80–84. 10.1016/j.neulet.2015.06.004 26057342

[B2] Amatriain-Fernandez S. Garcia-Noblejas M. E. Budde H. (2021). Effects of chronic exercise on the inhibitory control of children and adolescents: a systematic review and meta-analysis. Scand. J. Med. Sci. Sports 31, 1196–1208. 10.1111/sms.13934 33559271

[B3] Ardila A. Bernal B. Rosselli M. (2018). Executive functions brain system: an activation likelihood estimation meta-analytic study. Arch. Clin. Neuropsychol. 33, 379–405. 10.1093/arclin/acx066 28961762

[B4] Aron A. R.. (2007). The neural basis of inhibition in cognitive control. Neuroscientist 13, 214–228. 10.1177/1073858407299288 17519365

[B5] Aron A. R.. (2010). From reactive to proactive and selective control: developing a richer model for stopping inappropriate responses. Biol. Psychiatry 69, e55–e68. 10.1016/j.biopsych.2010.07.024 20932513 PMC3039712

[B6] Arzimanoglou A. Aldenkamp A. Cross H. Lassonde M. (2005). Cognitive Dysfunction in Children with Temporal Lobe Epilepsy (Vol. 1). John Libbey Eurotext.

[B7] Bari A. Robbins T. W. W. (2013). Noradrenergic versus dopaminergic modulation of impulsivity, attention and monitoring behaviour in rats performing the stop-signal task. Psychopharmacology 230, 89–111. 10.1007/s00213-013-3141-6 23681165 PMC3824307

[B8] Baumeister S. Wolf I. Holz N. Boecker-Schlier R. Adamo N. Holtmann M. . (2018). Neurofeedback training effects on inhibitory brain activation in ADHD: a matter of learning? Neuroscience 378, 89–99. 10.1016/j.neuroscience.2016.09.025 27659116

[B9] Baym C. L. Corbett B. A. Wright S. B. Bunge S. A. (2008). Neural correlates of tic severity and cognitive control in children with Tourette syndrome. Brain 131, 165–179. 10.1093/brain/awm278 18056159

[B10] Biazus-Sehn L. F. Schuch F. B. Firth J. Stugger F. S. (2020). Effects of physical exercise on cognitive function of older adults with mild cognitive impairment: a systematic review and meta -analysis. Arch. Gerontol. Geriatr. 89:104048. 10.1016/j.archger.2020.104048 32460123

[B11] Brady K. T. Gray K. M. Tolliver B. K. (2011). Cognitive enhancers in the treatment of substance use disorders: clinical evidence. Pharmacol. Biochem. Behav. 99, 285–294. 10.1016/j.pbb.2011.04.017 21557964 PMC3114106

[B12] Brown M. W. Aggleton J. P. (2001). Recognition memory: what are the roles of the perirhinal cortex and hippocampus? Nat. Rev. Neurosci. 2, 51–61. 10.1038/35049064 11253359

[B13] Cabeza R. Nyberg L. (2000). Imaging cognition II: an empirical review of 275 PET and fMRI studies. J. Cogn. Neurosci. 12, 1–47. 10.1162/08989290051137585 10769304

[B14] Chen F. Hung T. M. Chang Y. (2021). Reply to: comment on: “effects of exercise training interventions on executive function in older adults: a systematic review and meta-analysis.” Sports Med. 51, 597–598. 10.1007/s40279-020-01370-0 33128736

[B15] Chu C. Alderman B. L. Wei G. Wei G. (2015). Effects of acute aerobic exercise on motor response inhibition: an ERP study using the stop-signal task. J. Sport Health Sci. 4, 73–81. 10.1016/j.jshs.2014.12.002

[B16] Colcombe S. J. Kramer A. F. Erickson K. I. Scalf P. McAuley E. Cohen N. J. . (2004). Cardiovascular fitness, cortical plasticity, and aging. Proc. Natl. Acad. Sci. U.S.A. 101, 3316–3321. 10.1073/pnas.0400266101 14978288 PMC373255

[B17] Coxon J. P. Van Impe A. Wenderoth N. Swinnen S. P. (2012). Aging and inhibitory control of action: cortico-subthalamic connection strength predicts stopping performance. J. Neurosci. 32, 8401–8412. 10.1523/JNEUROSCI.6360-11.2012 22699920 PMC6703643

[B18] Crosbie J. Perusse D. Barr C. L. Schachar R. J. (2008). Validating psychiatric endophenotypes: inhibitory control and attention deficit hyperactivity disorder. Neurosci. Biobehav. Rev. 32, 40–55. 10.1016/j.neubiorev.2007.05.002 17976721

[B19] Cui J. Zou L. Herold F. Yu Q. Jiao C. Zhang Y. . (2020). Does cardiorespiratory fitness influence the effect of acute aerobic exercise on executive function? Front. Hum. Neurosci. 14:569010. 10.3389/fnhum.2020.569010 33132882 PMC7573667

[B20] Enticott P. G. Ogloff J. R. Bradshaw J. L. (2008). Response inhibition and impulsivity in schizophrenia. Psychiatry Res. 157, 251–254. 10.1016/j.psychres.2007.04.007 17916385

[B21] Garavan H. Ross T. J. Murphy K. Roche R. A. Stein E. A. (2002). Dissociable executive functions in the dynamic control of behavior: inhibition, error detection, and correction. Neuroimage 17, 1820–1829. 10.1006/nimg.2002.1326 12498755

[B22] Haber S. N. Fudge J. L. McFarland N. R. (2000). Striatonigrostriatal pathways in primates form an ascending spiral from the shell to the dorsolateral striatum. J. Neurosci. 20, 2369–2382. 10.1523/JNEUROSCI.20-06-02369.2000 10704511 PMC6772499

[B23] Hidiroglu C. Torres I. J. Er A. Isik G. Yaln N. Yatham L. N. . (2015). Response inhibition and interference control in patients with bipolar I disorder and first-degree relatives. Bipolar Disord. 17, 781–794. 10.1111/bdi.12335 26415581

[B24] Hsu C. L. Best J. R. Davis J. C. Nagamatsu L. S. Wang S. Boyd L. A. . (2018). Aerobic exercise promotes executive functions and impacts functional neural activity among older adults with vascular cognitive impairment. Br. J. Sports Med. 52:184. 10.1136/bjsports-2016-096846 28432077

[B25] Hughes M. E. Fulham W. R. Johnston P. J. Michie P. T. (2012). Stop-signal response inhibition in schizophrenia: behavioural, event-related potential and functional neuroimaging data. Biol. Psychol. 89, 220–231. 10.1016/j.biopsycho.2011.10.013 22027085

[B26] Indovina I. Macaluso E. (2004). Occipital-parietal interactions during shifts of exogenous visuospatial attention: trial-dependent changes of effective connectivity. Magn. Reson. Imaging 22, 1477–1486. 10.1016/j.mri.2004.10.016 15707797

[B27] Jahfari S. Waldorp L. van den Wildenberg W. P. Scholte H. S. Ridderinkhof K. R. Forstmann B. U. (2011). Effective connectivity reveals important roles for both the hyperdirect (fronto-subthalamic) and the indirect (fronto-striatal-pallidal) fronto-basal ganglia pathways during response inhibition. J. Neurosci. 31, 6891–6899. 10.1523/JNEUROSCI.5253-10.2011 21543619 PMC6632844

[B28] Ji L. Steffens D. C. Wang L. (2021). Effects of physical exercise on the aging brain across imaging modalities: a meta-analysis of neuroimaging studies in randomized controlled trials. Int. J. Geriatr. Psychiatry 36, 1148–1157. 10.1002/gps.5510 33675074

[B29] Krafft C. E. Schwarz N. F. Chi L. Weinberger A. L. Schaeffer D. J. Pierce J. E. . (2014). An 8-month randomized controlled exercise trial alters brain activation during cognitive tasks in overweight children. Obesity 22, 232–242. 10.1002/oby.20518 23788510 PMC4077546

[B30] Kunishio K. Haber S. N. (1994). Primate cingulostriatal projection: limbic striatal versus sensorimotor striatal input. J. Comp. Neurol. 350, 337–356. 10.1002/cne.903500302 7533796

[B31] Laird A. R. Fox P. M. Price C. J. Glahn D. C. Uecker A. M. Lancaster J. L. . (2005). ALE meta-analysis: controlling the false discovery rate and performing statistical contrasts. Hum. Brain Mapp. 25, 155–164. 10.1002/hbm.20136 15846811 PMC6871747

[B32] Lee R. S. Hoppenbrouwers S. Franken I. (2015). A systematic meta-review of impulsivity and compulsivity in addictive behaviors. Neuropsychol. Rev. 29, 14–26. 10.1007/s11065-019-09402-x 30927147

[B33] Li L. Zhang J. Cao M. Hu W. Zhou T. Huang T. . (2020). The effects of chronic physical activity interventions on executive functions in children aged 3-7 years: a meta-analysis. J. Sci. Med. Sport 23, 949–954. 10.1016/j.jsams.2020.03.007 32360243

[B34] Li M. Y. Huang M. M. Li S. Z. Tao J. Zheng G. Chen L. (2017). The effects of aerobic exercise on the structure and function of DMN-related brain regions: a systematic review. Int. J. Neurosci. 127, 634–649. 10.1080/00207454.2016.1212855 27412353

[B35] Liang X. Li R. Wong S. H. Sum R. K. Sit C. H. P. (2021a). The impact of exercise interventions concerning executive functions of children and adolescents with attention-deficit/hyperactive disorder: a systematic review and meta-analysis. Int. J. Behav. Nutr. Phys. Act 18:68. 10.1186/s12966-021-01135-6 34022908 PMC8141166

[B36] Liang X. Li R. Wong S. H. Sum R. K. Wang P. Yang B. . (2021b). The effects of exercise interventions on executive functions in children and adolescents with autism spectrum disorder: a systematic review and meta-analysis. Sports Med. 52, 75–88. 10.1007/s40279-021-01545-3 34468951

[B37] Liao D. Huang C. Hu S. Fang S. Wu C. Chen W. . (2014). Cognitive control in opioid dependence and methadone maintenance treatment. PLoS ONE 9:e94589. 10.1371/journal.pone.0094589 24727743 PMC3984179

[B38] Liu-Ambrose T. Nagamatsu L. S. Voss M. W. Khan K. M. Handy T. C. (2012). Resistance training and functional plasticity of the aging brain: a 12-month randomized controlled trial. Neurobiol. Aging 33, 1690–1698. 10.1016/j.neurobiolaging.2011.05.010 21741129

[B39] Loprinzi P. D.. (2017). The effects of physical exercise on parahippocampal function. Physiol. Int. 106, 114–127. 10.1556/2060.106.2019.10 31282762

[B40] Loprinzi P. D. Herod S. M. Cardinal B. J. Noakes T. D. (2013). Physical activity and the brain: a review of this dynamic, bi-directional relationship. Brain Res. 1539, 95–104. 10.1016/j.brainres.2013.10.004 24120986

[B41] Martinsen S. Flodin P. Berrebi J. Löfgren M. Bileviciute-Ljungar I. Mannerkorpi K. . (2018). The role of long-term physical exercise on performance and brain activation during the Stroop colour word task in fibromyalgia patients. Clin. Physiol. Funct. Imaging 38, 508–516. 10.1111/cpf.12449 28627125

[B42] Mehren A. Luque C. D. Brandes M. Lam A. P. Thiel C. M. Philipsen A. . (2019a). Intensity-dependent effects of acute exercise on executive function. Neural Plast 2019:8608317. 10.1155/2019/8608317 31281346 PMC6589258

[B43] Mehren A. Oezyurt J. Lam A. P. Brandes M. Müller H. H. Thiel C. M. . (2019b). Acute effects of aerobic exercise on executive function and attention in adult patients with ADHD. Front. Psychiatry 10:132. 10.3389/fpsyt.2019.00132 30971959 PMC6443849

[B44] Mehren A. Ozyurt J. Thiel C. M. Brandes M. Lam A. P. Philipsen A. (2019c). Effects of acute aerobic exercise on response inhibition in adult patients with ADHD. Sci. Rep. 9:19884. 10.1038/s41598-019-56332-y 31882652 PMC6934617

[B45] Meng X. Huang D. Ao H. Wang X. Gao X. (2020). Food cue recruits increased reward processing and decreased inhibitory control processing in the obese/overweight: an activation C likelihood estimation meta-analysis of fMRI studies. Obes. Res. Clin. Pract. 14, 127–135. 10.1016/j.orcp.2020.02.004 32098756

[B46] Metcalfe A. W. MacIntosh B. J. Scavone A. Ou X. Korczak D. Goldstein B. I. (2016). Effects of acute aerobic exercise on neural correlates of attention and inhibition in adolescents with bipolar disorder. Transl. Psychiatry 6:e814. 10.1038/tp.2016.85 27187236 PMC5070058

[B47] Muller P. Rehfeld K. Schmicker M. Hökelmann A. Dordevic M. Lessmann V. . (2017). Evolution of neuroplasticity in response to physical activity in old age: the case for dancing. Front. Aging Neurosci. 9:56. 10.3389/fnagi.2017.00056 28352225 PMC5348543

[B48] Nachev P. Kennard C. Husain M. (2008). Functional role of the supplementary and pre-supplementary motor areas. Nat. Rev. Neurosci. 9, 856–869. 10.1038/nrn2478 18843271

[B49] Nakahara K. Hayashi T. Konishi S. Miyashita Y. (2002). Functional MRI of macaque monkeys performing a cognitive set-shifting task. Science. 295, 1532–1536. 10.1126/science.1067653 11859197

[B50] Niendam T. A. Laird A. R. Ray K. L. Dean Y. M. Glahn D. C. Carter C. S. (2012). Meta-analytic evidence for a superordinate cognitive control network subserving diverse executive functions. Cogn. Affect. Behav. Neurosci. 12, 241–268. 10.3758/s13415-011-0083-5 22282036 PMC3660731

[B51] Pensel M. C. Daamen M. Scheef L. Knigge H. U. Vega S. R. Martin J. A. . (2018). Executive control processes are associated with individual fitness outcomes following regular exercise training: blood lactate profile curves and neuroimaging findings. Sci. Rep. 8:4893. 10.1038/s41598-018-23308-3 29559674 PMC5861091

[B52] Poldrack R. A. Fletcher P. C. Henson R. N Worsley K. J. Brett M. Nichols T. E. (2008). Guidelines for reporting an fMRI study. Neuroimage 40, 409–414. 10.1016/j.neuroimage.2007.11.048 18191585 PMC2287206

[B53] Raslau F. D. Mark I. T. Klein A. P. Ulmer J. Mathews V. Mark L. P. (2015). Memory part 2: the role of the medial temporal lobe. Am. J. Neuroradiol. 36, 846–849. 10.3174/ajnr.A4169 25414002 PMC7990589

[B54] Robinson J. L. Laird A. R. Glahn D. C. Lovallo W. R. Fox P. T. (2010). Metaanalytic connectivity modeling: delineating the functional connectivity of the human amygdala. Hum. Brain Mapp. 31, 173–184. 10.1002/hbm.20854 19603407 PMC2872058

[B55] Sachs M. Kaplan J. Sarkissian A. D. Habibi A. (2017). Increased engagement of the cognitive control network associated with music training in children during an fMRI Stroop task. PLoS ONE 12:e0187254. 10.1371/journal.pone.0187254 29084283 PMC5662181

[B56] Sherrington C. Herbert R. D. Maher C. G. Moseley A. M. (2000). PEDro. A database of randomized trials and. systematic reviews in physiotherapy. Man Ther. 5, 223–226. 10.1054/math.2000.0372 11052901

[B57] Smittenaar P. Rutledge R. B. Zeidman P. Adams R. A. Brown H. Lewis G. . (2015). Proactive and reactive response inhibition across the lifespan. PLoS ONE 10:e0140383. 10.1371/journal.pone.0140383 26488166 PMC4619547

[B58] Sofuoglu M. DeVito E. E. Waters A. J. Carroll K. M. (2013). Cognitive enhancement as a treatment for drug addictions. Neuropharmacology 64, 452–463. 10.1016/j.neuropharm.2012.06.021 22735770 PMC3445733

[B59] Turkeltaub P. E. Eden G. F. Jones K. M. Zeffiro T. A. (2002). Meta-analysis of the functional neuroanatomy of single-word reading: method and validation. Neuroimage 16, 765–780. 10.1006/nimg.2002.1131 12169260

[B60] Won J. Alfini A. J. Weiss L. R. Callow D. D. Smith J. C. (2019). Brain activation during executive control after acute exercise in older adults. Int. J. Psychophysiol. 146, 240–248. 10.1016/j.ijpsycho.2019.10.002 31639380

[B61] Wu M. Tang P. Goh J. O. Chou T. Chang Y. Hsu Y. . (2018). Task-switching performance improvements after tai chi chuan training are associated with greater prefrontal activation in older adults. Front. Aging Neurosci. 10:280. 10.3389/fnagi.2018.00280 30319391 PMC6165861

[B62] Yarkoni T. Poldrack R. A. Nichols T. E. Van Essen D. C. Wager T. D. (2011). Large-scale automated synthesis of human functional neuroimaging data. Nat. Methods 8, 665–670. 10.1038/nmeth.1635 21706013 PMC3146590

[B63] Yeo B. T. Krienen F. M. Sepulcre J. Sabuncu M. R. Lashkari D. Hollinshead M. . (2011). The organization of the human cerebral cortex estimated by intrinsic functional connectivity. J. Neurophysiol. 106, 1125–1165. 10.1152/jn.00338.2011 21653723 PMC3174820

[B64] Yu Q. Herold F. Becker B. Klugah-Brown B. Zhang Y. Perrey S. . (2021). Cognitive benefits of exercise interventions: an fMRI activation likelihood estimation meta-analysis. Brain Struct. Funct. 226, 601–619. 10.1007/s00429-021-02247-2 33675397

