# Supplementary material for: Effects of Exercise on Neural Changes in Inhibitory Control: An ALE Meta-Analysis of fMRI Studies
Source: Front Hum Neurosci. 2022 Jun 24;16:891095. doi: 10.3389/fnhum.2022.891095 (PMC9265250; doi:10.3389/fnhum.2022.891095)
Supplement: Supplementary file 1 [file Table_1.docx]

| Database | Search strategy | Limits |
| --- | --- | --- |
| PsyclNFO: 293 (By using abstract)  Pubmed: 423 (By using title/abstract) Scopus: 1504 (By using title/abstract/key)  Web of science: 1864 (By using topic) | (“physical activit*” OR “physical fitness” OR “physical exercise” OR “physical education” OR “leisure activit*” OR “motor activit*” OR “sport* participation” OR fitness OR “cardiovascular fitness” OR exercis* OR “acute exercise” OR “chronic exercise” OR “healthy exercise” OR “aerobic exercise” OR“aerobic training” OR “exercise interval training” OR coordinative exercise” OR “coordinative training” OR “plyometric exercise” OR “plyometric training” OR “resistance exercise” OR “resistance training” OR“strength exercise” OR “strength training” OR “musculoskeletal intervention” OR “functional training” OR “motor learning”OR “motor skills” OR physiotherapy OR baseball OR basketball OR movement OR stretching OR bicycling OR boxing OR football OR golf OR gymnastics OR hockey OR “Tai Ji” OR mountaineering OR “racquet sports” OR tennis OR skating OR “snow sports skiing” OR soccer OR “sports for persons with disabilities” OR“ track and field” OR “youth sports” OR wrestling OR “weight lifting” OR “games recreational” OR “motor intervention” OR braces OR “behavior skills training ”OR “task specific training” OR “weight bearing exercise” OR running OR agility OR swimming OR “aquatic exercise” OR “horse riding” OR trampoline OR snowshoeing OR skating OR exergaming OR skateboarding OR dance OR walking OR treadmill) AND (“executive function” OR “executive dysfunction” OR “inhibitory control” OR self-control OR “behavioral inhibition” OR “cognitive control” OR“response inhibition” OR “interference control” OR “response inhibition” OR “impulse control”OR inhibit*OR “Go/No go” OR stop-signal OR “continuous Performance test” OR “sustained attention to response task”OR“hayling task”OR stroop OR Flanker OR “Simon tasks” ) AND (fMRI OR “functional MRI” OR “functional magnetic resonance imaging”) | -Human;  -English language |
|  |  |  |
|  |  |  |

**Appendix Table.** The detailed search strategy
